# Arene Ru(II) Complexes Acted as Potential *KRAS* G-Quadruplex DNA Stabilizer Induced DNA Damage Mediated Apoptosis to Inhibit Breast Cancer Progress

**DOI:** 10.3390/molecules27103046

**Published:** 2022-05-10

**Authors:** Jiayi Qian, Ruotong Liu, Ningzhi Liu, Chanling Yuan, Qiong Wu, Yanhua Chen, Weijun Tan, Wenjie Mei

**Affiliations:** 1 School of Pharmacy, Guangdong Pharmaceutical University, Guangzhou 510006, China; qianjiayi0101@163.com (J.Q.); ruotongliu123@126.com (R.L.); ganoderma0@126.com (N.L.); ycc2552@163.com (C.Y.); amandachen423@163.com (Y.C.); 2 Guangdong Province Engineering Technology Centre for Molecular Probes and Biomedicine Imaging, Guangzhou 510006, China; wuqiongniu.1113@163.com; 3 School of Food Science, Guangdong Pharmaceutical University, Guangzhou 510006, China

**Keywords:** arene ruthenium complex, *KRAS* G-quadruplex DNA, breast cancer, apoptosis, DNA damage

## Abstract

A series of arene Ru(II) complexes, [(*η*^6^-MeC_6_H_5_)Ru(L)Cl]Cl, (L=*o*-ClPIP, **1**; *m*-ClPIP, **2** and *p*-ClPIP, **3**) (*o*-ClPIP=2-(2-chlorophenyl)imidazo[4,5-f][1,10]phenanthroline; *m*-ClPIP=2-(3-chlorophenyl)imidazo[4,5-f][1,10]phenanthroline; *p*-ClPIP=2-(4-chlorophenyl)imidazo[4,5-f][1,10]phenanthroline) was synthesized and investigated as a potential apoptosis inducer in chemotherapy. Spectroscopy and molecular docking simulations show that **1** exhibits moderated binding affinity to *KRAS* G-quadruplex DNA by groove mode. Further, in vitro studies reveal that **1** displays inhibitory activity against MCF-7 growth with IC_50_ = 3.7 ± 0.2 μM. Flow cytometric analysis, comet assay, and immunofluorescence confirm that **1** can induce the apoptosis of MCF-7 cells and G0/G1 phase arrest through DNA damage. In summary, the prepared arene Ru(II) complexes can be developed as a promising candidate for targeting G-quadruplex structure to induce the apoptosis of breast cancer cells via binding and stabilizing *KRAS* G-quadruplex conformation on oncogene promoter.

## 1. Introduction

Arene Ru(II) complexes displayed diverse biological properties, such as anti-parasitic, anti-oxidant, anti-bacterial, and anti-cancer activities, and it has been regarded as the most promising platinum-based anti-tumor candidate [1,2,3]. In recent years, the biological function of a number of arene Ru(II) complexes have attracted widespread attention, their excellent inhibitory effect against different tumor cells in vitro and in vivo, as well as great DNA/RNA binding behaviors, have been extensively reported [4,5,6]. Nhukeaw et al. reported that RAPTA-T could inhibit the proliferation of BRCA1-defective HCC1937 breast cancer cells [7]. Hager et al. showed that arene Ru(II) complexes bearing naphthyl-substitution were cytotoxic to ovarian cancer cells [8]. Rahman and co-workers stated that bis-salicylaldimine-based dimetallic Ru-(*p*-cymene) complexes induced cancer cell apoptosis through the P53 pathway [9]. Arene Ru(II) complexes containing ferrocenamide ligands can also inhibit the proliferation of various tumor cells [10]. Arene Ru(II) complexes bearing benzamide derivatives exhibit good DNA binding behavior and anti-cancer effects [11,12]. The results consistently suggest these complexes as prospective metal drugs for cancer chemotherapy. In our previous studies, arene Ru(II) complexes coordinated by phenanthroimidazole derivates displayed remarkable anti-tumor activity and low toxicity to normal cells [13,14,15]. Further investigation showed that this kind of arene Ru(II) complexes could suppress the proliferation, migration, and invasion of cancer cells by blocking the formation of invadopodia via inducing DNA damage and S-phase arrest [16,17]. In addition, some of these arene Ru(II) complexes exhibit promising affinity to G-quadruplex DNA [18,19,20,21]. Given that G-quadruplex DNA regulates tumorigenesis, arene Ru(II) complexes may have good prospects in chemotherapy [8,22].

The *KRAS* gene is defined as an oncogene from Ras family; the mutation of the *KRAS* gene has the potential to become cancerous [23,24]. The *KRAS* protein is a membrane-bound protein which could bind to GTP to activate intracellular signaling pathways, promote tumor cells proliferation, and angiogenesis [25,26]. Moreover, the proliferation and metastasis of multiple tumors, including breast cancer, can be inhibited by different small molecules regulating the expression of *KRAS* [25,27,28]. A regular G-rich sequence (G_≥3_N_x_G_≥3_N_x_G_≥3_N_x_G_≥3_) found in the promoter of *KRAS* gene can be physically folded into G-quadruplex formation [29]. Accumulating evidence revealed the potential anti-tumor activity of small molecules targeting *KRAS* G-quadruplex DNA molecules. For example, natural products [29], porphyrin derivatives [30], quinoline derivatives [31], and aromatic compounds [32] are considered promising *KRAS* G-quadruplex stabilizers. A major research breakthrough has been achieved using a small molecule AMG 510 as a potential mutant *KRAS*(G12C) inhibitor with high specificity and sensitivity to effectively block tumor growth in vivo [33]. However, the inhibition of tumor cell proliferation through the binding interactions between arene Ru(II) complexes and *KRAS* G-quadruplex DNA is rarely examined. Hence, the development of a *KRAS* G-quadruplex DNA binding agent is a new strategy to inhibit the growth of breast cancer cells.

In this study, a series of arene Ru(II) complexes (**1**, **2**, and **3**) was synthesized through microwave irradiation. The interaction of these complexes with *KRAS* G-quadruplex DNA was studied by spectroscopy and molecular docking simulations, and their inhibitory activity against various human tumor cells was evaluated by MTT assay. Flow cytometry, comet assay, and immunofluorescence analysis confirmed that these arene Ru(II) complexes, especially **1,** can inhibit the growth of MCF-7 by inducing the apoptosis of tumor cells via DNA damage and G0/G1 cell cycle arrest.

## 2. Results

### 2.1. Synthesis and Characterization

**Scheme 1:**
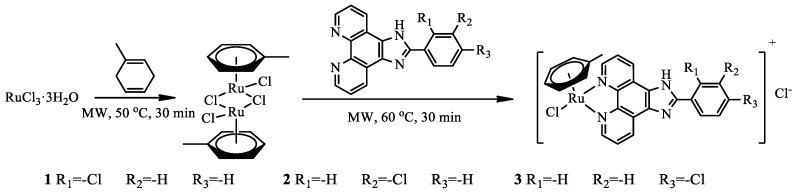
The synthetic route of arene Ru(II) complexes **1**, **2**, and **3**.

The position and steric hindrance of substituted groups in the terminal phenyl ring are crucial in the biological activities of drugs [34,35]. Initial SAR analysis on arene Ru(II) phenanthroimidazole derivatives revealed that the presence of halogens at the phenyl ring resulted in the production of the most potent compounds [19]. Therefore, the focus of this study was chloride substitution at different positions to generate complexes with ortho-, meta-, and para-substituted phenanthroimidazole ligands. Targets **1**–**3** were prepared using [(*η*^6^-CH_3_C_6_H_5_)RuCl_2_]Cl_2_ and imidazo[4,5-f][1,10]phenanthroimidazole modified with different substituted position groups with average yields higher than 90% under microwave irradiation at 60 °C for 30 min (Scheme 1).

For a long time, the role of the halogen bond (X bonding) in drug development has attracted widespread attention. It refers to the highly oriented attraction of the halogen atom to the electron donor, which is conducive to the interaction with the drug target [36]. The previous studies reported that this class of arene Ru(II) complexes modified with different halogen-substituents displayed better anti-tumor activity and stronger G-quadruplex DNA affinity than no halogen-substituents, especially for Cl-substituents and Br-substituents [20]. In order to research the complexes with favorable effectiveness and low toxicity, the Cl element was introduced in the structure of arene Ru(II) complexes to promote their biological effects.

The targeted complexes were characterized by ESI-MS, ^1^H NMR, and ^1^^3^C NMR spectroscopy (Figures S1–S9 in the Supplementary Materials). Figure S2A shows that the three peaks at chemical shift(δ) 9.94, 9.23, and 8.22 were attributed to 1, 3, and 2 position 2 H signals on the imidazophenanthroline of **1**, and 9.05 was attributed to 4 position H signal on the N atom of imidazole ring, respectively. The three peaks at δ 7.99–7.58 were attributed to the chemical shifts of 5, 6, 7, and 8 positions 4 H atoms on the terminal phenyl ring. The peaks at δ 6.46, 6.11, 5.91, and 2.29 were attributed to the chemical shifts of the b, c, a, and d position H atom on the phenyl ring of the aromatic ligand, respectively. Moreover, for complex **2** and **3**, the similar H signals assignments, there were a little difference of four H atoms on the terminal phenyl rings (Figures S5 and S8). Moreover, the HPLC results showed that the purity of three complexes was higher than 95% in the mobile phase of CH_3_OH:CH_3_CN=8:2 (Figures S10–S12).

### 2.2. Theoretical Calculation

**Figure 1:**
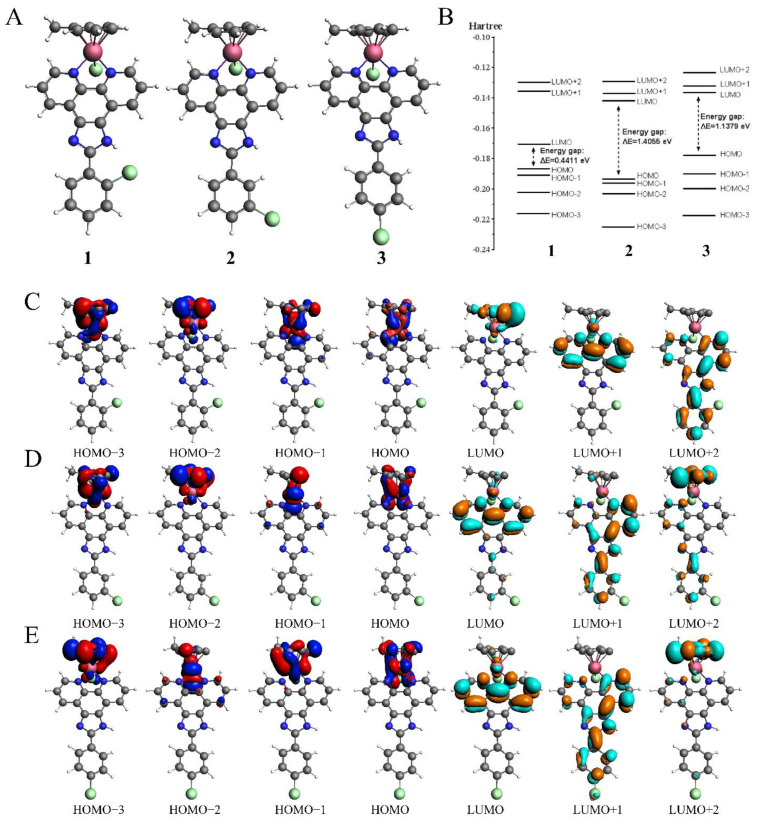
(**A**) Molecular structures of **1**, **2**, and **3**. (**B**) Gas-phase highest occupied molecular orbital (HOMO) and lowest unoccupied molecular orbital (LUMO) energies of **1**, **2**, and **3** (in hartree). (**C**) HOMOs (first four) and LUMOs (last three) of **1** (**C**), **2** (**D**), and **3** (**E**).

The electron configuration and orbital energies of three complexes were studied to analyze the probable cause under the binding of these three molecules with G-quadruplex DNA. The electric shell structure of the ground state and conduct orbital binding analyses of **1**, **2**, and **3** in accordance with a previously published methodology were calculated by using the ADF package (2019.10.4, SCM). In particular, GGA-BP86 and scalar relativistic corrections were adopted for structure optimization of the three molecular structures (Figure 1A). The ground state of Ru(II) for **1**, **2**, and **3** was a single state (S ¼ 0) without unpaired electrons in the three molecules, and the total bonding energies were −7240.98, −7274.23l and −7278.38 kcal/mol, respectively. Then, the electron density distribution of the molecular frontier orbital energy and the substitute group was further analyzed, and some dependencies were observed. The calculation results showed that **1** had a lower energy gap between HOMO and LUMO at 0.4411 eV than **2** and **3** at 1.4055 and 1.1379 eV (Figure 1B), because the electron intercalated and de-intercalated easily due to the smaller energy that the orbit leaped from the HOMO to the LUMO. These results indicated that the presence of Cl atom at the ortho-positionof the terminal phenyl ring in **1** could shift the LUMO of the molecule positively and reduce the force of electron injection into the conduction band. Moreover, for complex **1**, the electronic clouds majorly focused on the auxiliary ligand of phenyl ring, but a small quantity was found on the main ligand of phenanthroimidazole on HOMO and LUMO (Figure 1C). However, for complexes **2** and **3**, the electronic clouds were distributed primarily in the main ligand of phenanthroimidazole of HOMO and LUMO. It is speculated that the low energy gap and enriched electrons were beneficial to the stronger interaction of complex **1** with G-quadruplex DNA than that of **2** and **3**.

### 2.3. Molecular Recognition of KRAS G-Quadruplex DNA

#### 2.3.1. Electronic Titration Data Analysis

*KRAS* oncogene promoter is a guanine-rich(G_≥3_N_x_G_≥3_N_x_G_≥3_N_x_G_≥3_) DNA sequence that can form a G-quadruplex structure. The G-quadruplex structure of the stable promoter region can participate in the transcriptional regulation of *KRAS*. Considering that the *KRAS* oncogene plays a key role in the pathogenesis, progression, and growth of breast cancer cells, the interaction of complex **1** with *KRAS* G-quadruplex DNA may serve as a promising target for anti-breast cancer [37].

**Figure 2:**
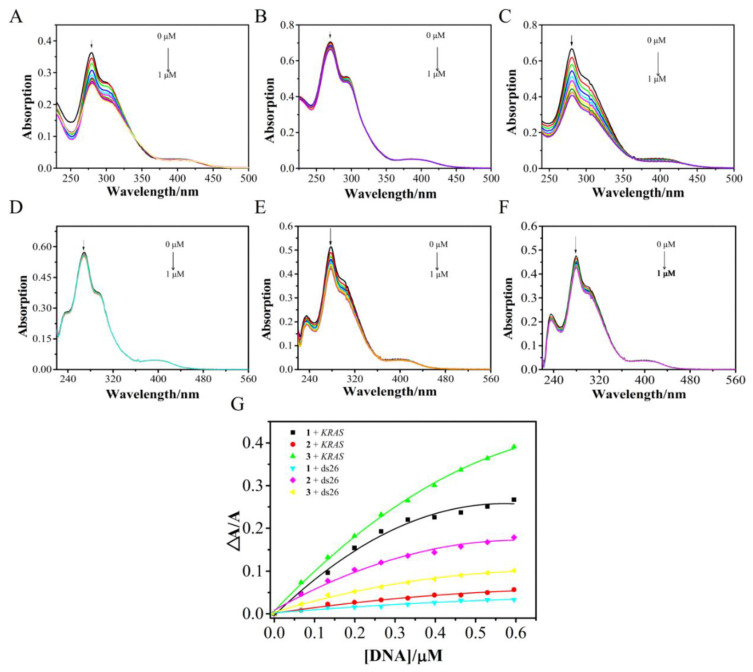
The electronic absorption spectra of complexes **1**, **2**, and **3** interacting with (**A**–**C**) *KRAS* G-quadruplex DNA; and (**D**–**F**) ds26 DNA. (**G**) ∆A/A vs. [DNA], ∆A = A_0_ − A.

**Figure 3:**
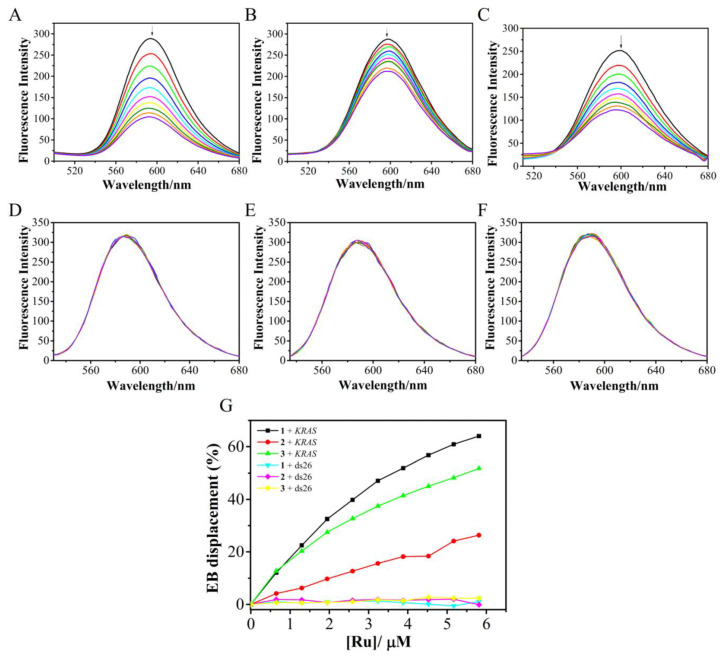
Emission spectra of ethidium bromide DNA (EB-DNA) in the incubation buffer in the absence and presence of complexes. (**A**–**C**) EB-*KRAS* G4 DNA and (**D**–**F**) EB-ds26 DNA. [EB] = 16 μM, [ds26 DNA] = 2 μM, [*KRAS* G4 DNA] = 2 μM. (**G**) Changes in EB displacement rate with the increasing concentrations of complexes in the EB-DNA system.

Electronic absorption spectroscopy was first conducted to investigate the binding behavior of the prepared complexes with the *KRAS* G-quadruplex DNA (Figure 2). Ru(II) complexes have unique spectral properties and exhibit color change and redshift in the presence of DNA. The degree of change is usually proportional to the binding strength. The intramolecular *KRAS* G-quadruplex structure was prepared in 10 mM Tris-HCl buffer (pH 7.4) with 100 mM KCl heated to 95 °C for 5 min, then cooled to room temperature overnight, and stored at 4 °C for 24 h. All three complexes exhibit characterized intraligand (IL) absorption with the range of 250–300 nm and a maximum of 280 nm, 270 nm, and 280 nm, respectively. The characterized metal-to-ligand charge transfer absorption is in the range of 350–450 nm. Shoulder absorption is also observed for these complexes and can be attributed to ligand-to-metal charge transfer absorption. Upon the addition of *KRAS* G-quadruplex DNA, **1** and **3** show hypochromism at IL absorption at approximately 27.8 and 39%, respectively. The binding constant (K_b_) is approximately 24.5 and 6.09 × 10^7^ M^−1^ according to the decay IL absorption for **1** and **3**, respectively [38,39]. As shown in Figure 2D,F, **1** and **3** had less binding affinity with double-stranded DNA and exerted 19.67 and 6.92 fold lower selective affinity, respectively, to the *KRAS* G-quadruplex DNA than to ds26 DNA. By contrast, no substantial hypochromism and redshift are observed for **2** in *KRAS* G-quadruplex DNA. This phenomenon is possibly due to the chloride substituents on the meta-positions in phenanthroimidazole that inhibit the effective intercalation of complex between the DNA base pairs [40]. Moreover, compound **2** showed minimal selectivity for G-quadruplex DNA. The position of the substituent may play an important role in the stability of G-quadruplex DNA.

#### 2.3.2. EB Displacement Assay

Given that almost all of these arene Ru(II) complexes do not emit florescence in Tris-HCl buffer solutions, their binding with *KRAS* G-quadruplex DNA and ds26 DNA was further confirmed by fluorescence quenching experiment. As fluorescence probe, EB emits strong fluorescence because it strongly intercalates within the base pairs of DNA. In addition, EB dissociates from DNA into the solvent system, thus reducing the fluorescence intensity of the EB–DNA system. As shown in Figure 3, the EB-DNA solution emits strong fluorescence at 500–700 nm, and the maximum fluorescence appears at 597 nm. Figure 3A–C shows that, with the successive addition of the complexes, the emission intensity of EB–DNA system is decreased, thus implying the displacement of EB from the hydrophobic pockets of *KRAS* G-quadruplex DNA as induced by the complex. With the addition of **1**, **2**, and **3**, the fluorescence intensity of the solution gradually decreases. The EB displacement rates (I_0_ − I)/I_0_ for *KRAS* G-quadruplex DNA were 64.0%, 26.4%, and 51.3% for **1**, **2**, and **3**. Double-stranded DNA was also tested by complexes, as shown in Figure 3D–F. Data on florescence emission intensity revealed minimal change for EB- ds26 DNA system with the increasing amount of the complexes. These results indicated that the arene Ru(II) derivatives exhibited great selectivity to the *KRAS* G-quadruplex structure, with **1** showing the best interaction.

#### 2.3.3. Molecular Docking

**Figure 4:**
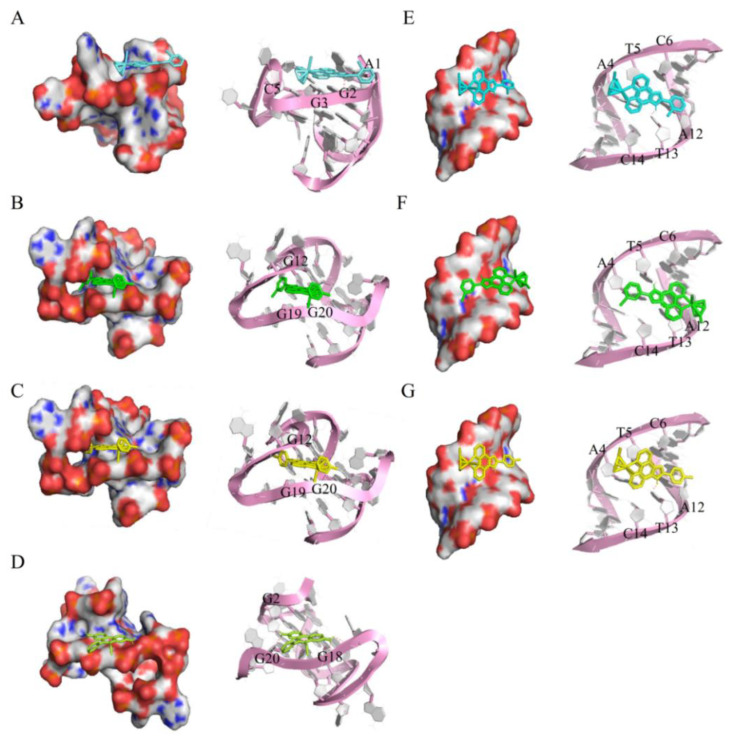
Binding mode of **1** (**A**), **2** (**B**), **3** (**C**), and EB (**D**) with *KRAS* G-quadruplex DNA and dsDNA (**E**–**G**) calculated by molecular docking.

Molecular docking simulation was performed to analyze the binding strength and mode between Ru(II) complexes and *KRAS* G-quadruplex DNA [20]. The structure of **1**, **2**, and **3** were optimized using the density functional theory (DFT) calculations. The results revealed that **1**, **2**, and **3** could be combined with the groove of *KRAS* G-quadruplex DNA. AS show in Figure 4, compound **1** can be inserted into the groove by A1, G2, G3, and G5 by groove binding mode, and the H atom of imidazole ring form 1 H-bonds with the residues of G3. The binding energy of **1** is −6.78 kcal/mol, which represents good interaction with *KRAS* G-quadruplex DNA. According to the calculation results, **2** and **3** can be inserted into the groove of *KRAS* G-quadruplex DNA formed by base pairs G12, G18 and G19, G20. The H on the N atom of the **2**, **3** imidazole ring can form hydrogen bonds with A15 residues with binding energies of −7.13 and −7.23 kcal/mol. These data indicated that **1**, **2**, and **3** might bind to G-quadruplex in a groove binding mode. Moreover, **1** and EB have adjacent binding sites on *KRAS*. This finding explains why **1** has the strongest EB displacement ability among the complexes during the fluorescence quenching experiment. The compounds were also docked with dsDNA for the comparative study. The results revealed that the compounds did not enter the intercalation site but rather electrostatically interacted with dsDNA. The energy of complexes **1**, **2**, and **3** to dsDNA were −6.68, −7.02, and −6.8 kcal/mol, respectively. These simulation results further indicated that this class of arene Ru(II) derivatives could selectively bind with *KRAS* G-quadruplex DNA more strongly than with duplex DNA. The arene Ru(II) derivatives could bind to *KRAS* G-quadruplex DNA, with **1** exhibiting the greatest selectivity.

### 2.4. Inhibiting the Growth of Breast Cancer Cells through DNA Damage Mediated Apoptosis

#### 2.4.1. Evaluation of Anti-Cancer Activity and Drug Uptake in Cell Culture

The inhibitory effect of these complexes against the growth of various tumor cell lines including breast cancer MDA-MB-231, MCF-7, esophageal cancer EC-1, and breast epithelial MCF-10A cells was evaluated by MTT assay [41], with concentrations inducing 50% cell growth inhibition (IC_50_ ± SD), which are listed in Table 1. After 72 h treatment with different concentrations of **1**, **2**, and **3**, all three complexes show inhibitory activity against tumor growth. The anti-proliferation activities (half-maximal inhibitory concentration) of complexes **1** against human breast cancer MCF-7 cells was approximately 3.7 μM. In contrast, **3** showed very low cytotoxicity to both MCF-7 and EC-1 cells. However, no cytotoxic effect was observed for each of the compounds in breast epithelial MCF-10A cell lines even up to 100 μM, indicating their selective effectiveness toward cancer cells rather than normal cells. These results indicated that the ortho-chloro-substituted arene Ru(II) complex has a good inhibitory effect on MCF-7 as having the lowest IC_50_ values compared to other compounds.

In order to clarify the relationship between the accumulation of complexes in tumor cells and inhibitory effect, the intracellular accumulation of the compounds was measured in three tumor cell lines. The cells were treated with **1**, **2**, **3** (20 μM) for 4 h, lysed with nitric acid (60%), and then diluted. Inductively Coupled Plasma (ICP)-MS was used to determine the content of ruthenium in both breast cancer cell line and breast epithelial cells. The rate at which the arene ruthenium complexes are absorbed by cells depends on their structural modification. As shown in Table 2, compound **2** showed the highest internalization in the three cell lines. These results indicated that the meta-substituent modification significantly enhances the accumulation of compounds in tumor cells. On the other hand, the cytotoxicity of a compound is affected by two elements, including the ability of the compound to be absorbed into the cell and the strength of the interaction between the compound and the target. In summary, although compound **2** showed a high degree of internalization in all three types of cells, its interaction with the target was weak; thus, it did not show effective cytotoxicity. Compound **1** showed a moderate degree of internalization in MCF-7 cells and an effective combination of *KRAS* G-quadruplex DNA, thus exhibiting obvious tumor suppressor activity.

#### 2.4.2. Apoptosis Induced through DNA Damage

**Figure 5:**
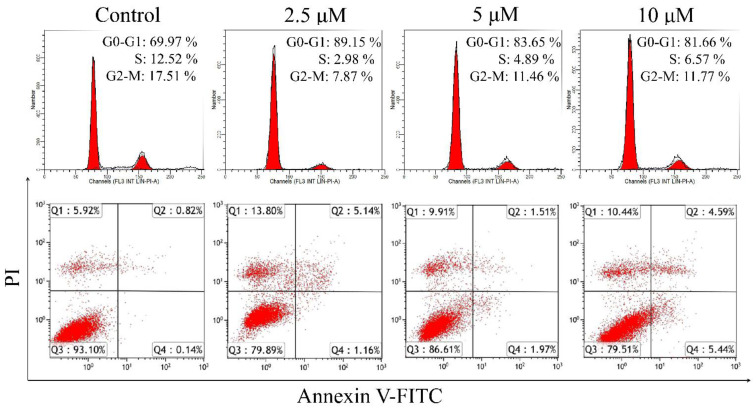
Apoptosis and cell cycle analysis of MCF-7 human breast cancer cells after 72 h of exposure to complexes **1** by flow cytometry using Annexin V-FITC vs. PI staining.

Cell cycle and apoptosis are the main reasons why organometallic compounds inhibit cancer cell growth [42,43]. In this work, flow cytometry was adopted. Figure 5 shows the MCF-7 cells treated with different concentrations of **1** after 72 h. In the high concentration treatment group, the portion of apoptosis cells is approximately 10.03%, which is 10 times higher than that in the control group (0.96%) [44,45]. In addition, treatment with **1** resulted in an increase in the distribution of cells in G0/G1 phase, with a decrease in S phase. The results suggest that one of the mechanisms of the growth inhibitory effect of **1** on breast cancer cells is mediated through cell-cycle arrest and apoptosis.

**Figure 6:**
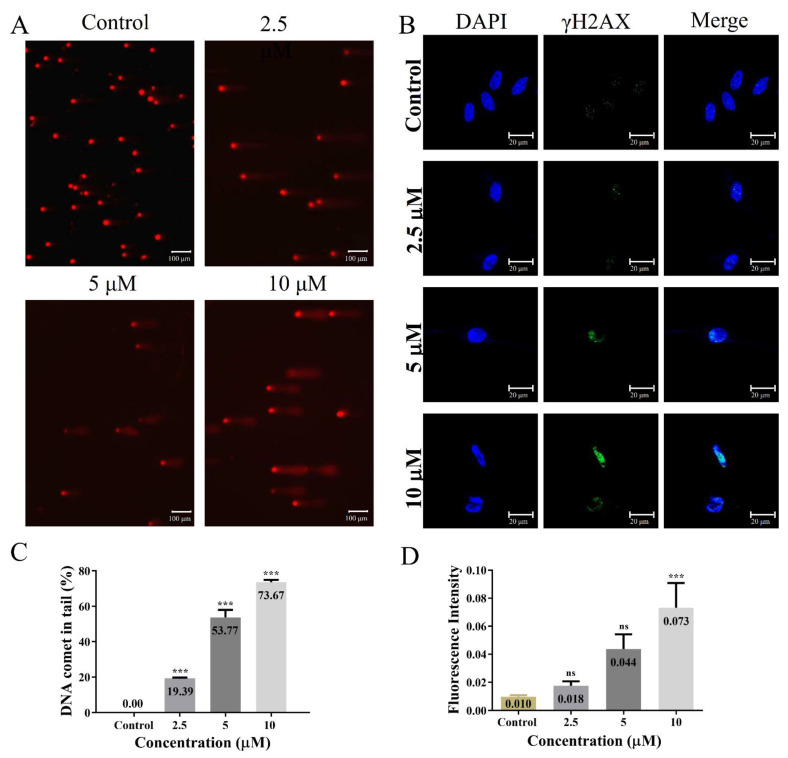
Elevated levels of DNA damage in MCF-7 Human breast cancer cells treated with complex **1**. (**A**) Representative images of MCF-7 cells treated with complex **1** (0, 2.5, 5, and 10 μM) for 72 h for comet assays. (**B**) Quantification of the tail moment using comet assays of cells treated as in (**A**); *n* = 3; error bars, SD. *p* values were calculated using an unpaired two-tailed *t* test. (**C**) Representative images of MCF-7 cells treated with **1** (0, 2.5, 5, and 10 μM) for 72 h before processing for immunofluorescence staining with anti-*γ*H2AX antibody (green). DNA was counterstained with DAPI (blue). (**D**) Corresponding quantification of (C) shows the normalized mean fluorescence intensity of γH2AX within the nucleus. (*** *p* < 0.001; ns represents not significant).

In this context, the amount of DNA damage was assessed in blank control and **1** (2.5, 5 and 10 μM) treated MCF-7 cell lines by single-cell gel electrophoresis and immunofluorescence. Figure 6A represents the photomicrograph of the comet assay. In the experiment, damaged DNA travels from the nucleus to the anode during electrophoresis, forming the shape of a “comet” with a head (the head contains the nucleus of intact DNA) and a tail (the DNA that has been released or broken). The assay is capable of detecting single-strand DNA breaks [46,47]. A substantial enhancement in the level of DNA damage was observed in MCF-7 cells after one treatment. Treatment with **1** at various concentrations can dose-dependently induce DNA damage in MCF-7 cells. The percentage of DNA in the tail were further examined via CASP software, of which 10 μM of **1** showed a maximum percentage of DNA in the tail (73%). Specific immunodetection the phosphorylation of the variant histone H2AX(γH2AX), is a highly sensitive method to monitor DSBs formation. After treatment with **1**, the proportion of γH2AX focus positive cells in MCF-7 cells was increased (Figure 6C). Therefore, DNA damage is the main cause of G0/G1 phase arrest and apoptosis in MCF-7 cells induced by **1**.

## 3. Materials and Methods

### 3.1. Reagents and Materials

All reagents and solvents were used as purchased from commercial suppliers without further purification. Ruthenium (Ru) chloride hydrate (99.7%, Mitsuwa Chemicals, Shanghai, China), 1,10-Phenanthroline monohydrate (99.7%, Aladdin, Shanghai, China), 1-methyl-1,4-cyclohexadiene (99.7% Aladdin, Shanghai, China), 2-chlorobenzaldehyde (99.7%, Shanghai, China), 3-chlorobenzaldehyde (99.7%, Aladdin, Shanghai, China), 4-chlorobenzaldehyde (99.7%, Aladdin, Shanghai, China), 1,10-Phenanthroline-5,6-dione (97%, J&K Chemical, Beijing, China). The ligand of *o*-ClPIP, *m*-ClPIP, and *p*-ClPIP were prepared by using a similar method as in the literature [48]. The synthetic arene Ru(II) complexes [(*η*^6^-CH_3_C_6_H_5_)RuCl_2_]_2_ were prepared by Anton Paar GmbH monowave 300 [49]. ^1^H NMR spectra and ^13^C NMR spectra were recorded in DMSO-*d*^6^ on Bruker AVANCE III HD 500 MHz spectrometer and ESI-MS spectra were obtained in methanol on Thermo ScientificQ Exactive ESI-MS system (Agilent, Palo Alto, CA, USA). Elemental analysis for C, H, and N were carried out on an elementar vario MICRO cube. The electronic absorption spectra were recorded on a Shimadzu UV-2550 spectrophotometer. The fluorescence emission spectra were recorded on a RF-5301 fluorescence spectrophotometer.

### 3.2. Synthesis and Characterization

The ligand 1,10-phenanthroline-5,6-dione derivatives were prepared by a similar method according to previous literature we reported, with some modifications [48]. A solution containing 1,10-phenanthroline-5,6-dione (1.6 mmol. 347 mg), substituted benzaldehyde derivatives (1.6 mmol), NH_4_Ac (33 mmol, 2.53 g), and 20 mL of HAc was irradiated under microwave at 110 °C for 30 min. Then, added 20 mL distilled water and adjusted pH value to 7.0 at room temperature. The sediment was filtered and dried in a vacuum to obtain a yellow crude product. In the next step, the crude product was dissolved in a mixed solvent of absolute ethanol and chloroform and purified by column chromatography on a silica gel column (60–100 mesh), using absolute ethanol as the eluent.

The ruthenium precursors [(*η*^6^-CH_3_C_6_H_5_)RuCl_2_]_2_ were prepared according to the literature procedure [49]. A mixture of RuCl_3_·3H_2_O (1.65 g, 6.3 mmol), 1-methyl-1,4cyclohexadiene (4 mL), and 90% ethanol (20 mL) into a 30 mL quartz tube under microwave irradiation at 90 °C 30 min. After the reaction, it was cooled to room temperature, filtered with suction, the filter cake was rinsed with ethanol and water several times, and dried in a vacuum.

#### 3.2.1. Synthesis of (*η*^6^-MeC_6_H_5_)Ru(o-ClPIP)Cl]Cl (**1**)

A mixture of [(*η*^6^-CH_3_C_6_H_5_)RuCl_2_]_2_ (0.1 mmol, 52.9 mg), ligand *o*-ClPIP (0.2 mmol, 66.1 mg), and dichloromethane (20 mL) was dissolved in a quartz reaction tube, through microwave-assisted heating at 60 °C for 30 min. The reaction solution was cooled to room temperature and rotary evaporated to obtain a yellow crude product. In the next step, 10 mL of methanol and 10 mL of acetonitrile were added for ultrasonic dissolution and filtration, and the filtrate was spin-dried under reduced pressure to obtain 102.2 mg of an orange solid with a yield of 85.9%. ESI-MS (in MeOH, *m*/*z*): 595 (Cal.), 559 (Found for [M-Cl]^+^). Calculated for C_34_H_35_Cl_3_N_6_ORu (%): C 54.37, H 4.70, N 11.19; Found (%): C 54.70, H 4.24, N 11.42. (One molecule containing 1Cl^−^, 1 CH_3_CH_2_OCH_2_CH_3_, 2 CH_3_CN). ^1^H NMR (500 MHz, DMSO) δ 9.92 (dd, *J* = 5.2, 0.8 Hz, 2H), 7.99 (d, *J* = 1.8 Hz, 1H), 7.98 (d, *J* = 1.8 Hz, 1H), 7.72 (dd, *J* = 7.9, 1.3 Hz, 1H), 7.60 (dtd, *J* = 18.8, 7.4, 1.6 Hz, 2H), 6.46 (t, *J* = 6.0 Hz, 2H), 6.11 (d, *J* = 6.2 Hz, 2H), 5.99 (t, *J* = 5.9 Hz, 1H), 5.90 (t, *J* = 5.7 Hz, 1H), 5.71 (dd, *J* = 8.8, 5.8 Hz, 1H), 2.28 (s, 3H).^13^C NMR (126 MHz, DMSO) δ 154.58 (s), 150.88 (s), 132.74 (s), 132.54 (s), 132.22 (s), 130.93 (s), 129.65 (s), 128.02 (s), 106.07 (s), 105.80 (s), 90.53 (s), 89.94 (s), 85.24 (s), 83.54 (s), 82.63 (s), 80.56 (s), 19.46–19.46 (m), 19.28 (s). EA (C_26_H_19_Cl_2_N_4_Ru (%)): C 55.82, H 3.42, N 10.02; Found (%): C 54.70, H 4.24, N 11.42. (One molecule containing 1CH_3_CN, 1H_2_O).

#### 3.2.2. Synthesis of (*η*^6^-MeC_6_H_5_)Ru(m-ClPIP)Cl]Cl (**2**)

**2** was synthesized with [(*η*^6^-CH_3_C_6_H_5_)RuCl_2_]_2_ (0.1 mmol, 52.9 mg), ligand *m*-ClPIP (0.2 mmol, 66.1 mg) in dichloromethane (20 mL), heating at 60 °C with microwave assisted heating for 30 min. The reaction solution was cooled to room temperature and rotary evaporated to obtain a yellow crude product. In the next step, 30 mL of methanol was added for ultrasonic dissolution and filtration, and the filtrate was spin-dried under reduced pressure to obtain 102.2 mg of an orange solid with a yield of 85.9%. ESI-MS (in MeOH, *m*/*z*): 595 (Cal.), 559 (Found for [M-Cl]^+^). Calculated for C_27_H_29_Cl_3_N_4_O_4_Ru (%):C 47.62, H 4.29, N 8.23, Found (%): C 47.63, H 4.27, N 8.06. (One molecule containing 1Cl^−^,1 CH_3_OH, 3 H_2_O). ^1^H NMR (500 MHz, DMSO) δ 9.88 (d, *J* = 4.7 Hz, 2H), 8.49 (s, 1H), 8.42 (t, *J* = 8.2 Hz, 1H), 8.15 (dd, *J* = 7.9, 5.4 Hz, 2H), 7.60–7.52 (m, 2H), 6.51–6.44 (m, 2H), 6.10 (d, *J* = 6.1 Hz, 2H), 5.99 (t, *J* = 5.9 Hz, 1H), 5.92 (t, *J* = 5.6 Hz, 1H), 5.75–5.67 (m, 2H), 2.31 (d, *J* = 7.5 Hz, 3H). ^13^C NMR (126 MHz, DMSO) δ 154.41 (s), 151.67 (s), 143.92 (s), 134.29 (s), 131.41 (s), 130.27 (s), 126.65 (s), 126.49 (s), 125.72 (s), 106.06 (d, *J* = 2.6 Hz), 90.60 (s), 89.93 (s), 85.24 (s), 83.42 (s), 82.63 (s), 80.37 (s), 19.34 (s). EA (C_26_H_19_Cl_2_N_4_Ru (%)): C 55.82, H 3.42, N 10.02; Found (%): C 47.63, H 4.27, N 8.06. (One molecule containing 1CH_3_OH, 2H_2_O, 1CH_2_Cl_2_).

#### 3.2.3. Synthesis of (*η*^6^-MeC_6_H_5_)Ru(p-ClPIP)Cl]Cl (**3**)

**3** was synthesized with [(*η*^6^-CH_3_C_6_H_5_)RuCl_2_]_2_ (0.1 mmol, 52.9 mg), ligand *p*-ClPIP (0.2 mmol, 66.1 mg) in dichloromethane (20 mL), heating at 60 °C with microwave assisted heating for 30 min. The reaction solution was cooled to room temperature and rotary evaporated to obtain a yellow crude product. In the next step, 30 mL of methanol was added for ultrasonic dissolution and filtration, and the filtrate was spin-dried under reduced pressure to obtain 116.0 mg of an orange solid with a yield of 97.5%. ESI-MS (in MeOH, *m*/*z*): 561.1, ([M+H]^+^). Calculated for C_35_H_43_Cl_9_N_4_O_3_Ru (%): C 42.55, H 4.39, N 5.67; Found (%): C 42.08, H 4.07, N 5.70. (One molecule containing 1 Cl^−^, 1 CH_3_CH_2_OCH_2_CH_3_, 2 CH_3_OH, 3 CH_2_Cl_2_). ^1^H NMR (500 MHz, DMSO) δ 9.88 (d, *J* = 5.0 Hz, 2H), 8.47 (d, *J* = 8.5 Hz, 2H), 8.17 (s, 2H), 7.68 (d, *J* = 8.5 Hz, 2H), 6.46 (t, *J* = 5.9 Hz, 2H), 6.11 (t, *J* = 9.8 Hz, 2H), 5.99 (t, *J* = 5.8 Hz, 1H), 5.91 (t, *J* = 5.6 Hz, 1H), 5.71 (t, *J* = 7.0 Hz, 2H), 2.30 (s, 3H).^13^C NMR (126 MHz, DMSO) δ 154.42 (s), 152.08 (s), 135.35 (s), 129.65 (s), 128.91 (s), 128.74 (s), 126.53 (s), 106.06 (d, *J* = 4.4 Hz), 90.59 (s), 89.94 (s), 85.24 (s), 83.43 (s), 82.63 (s), 80.38 (s), 19.33 (s). EA (C_26_H_19_Cl_2_N_4_Ru (%)): C 55.82, H 3.42, N 10.02; Found (%): C 42.08, H 4.07, N 5.70. (One molecule containing 2 CH_3_CH_2_OH, 2 H_2_O, 3 CH_2_Cl_2_).

### 3.3. Electronic Absorption Titration

The electronic spectra were recorded, and the interaction of **1**, **2**, and **3** with *KRAS* G-quadruplex DNA was clarified. Ru(II) complex absorption titration in a Tris-HCl buffer was performed using a certain complex concentration with a DNA stock solution added. The concentration of the compound was 20 μM. The electronic absorption titration experiments were recorded at 200–800 nm with the increasing concentration of *KRAS* G-quadruplex DNA (from 0 to 0.6 μM). Each solution was kept for 3 min at room temperature to achieve equilibrium before detection. The titration process was repeated several times until no change in the spectrum indicating binding saturation was found. At the end of each titration, the changes in concentration of the diluted Ru(II) complex can be ignored. Analysis was performed by the following equation.

(ε_a_ − ε_f_)/(ε_b_ − ε_f_) = [b − (b^2^ − 2K^2^C_t_[DNA]/S)]^1/2^/2KC_t_

B = 1 + KC_t_ + K[DNA]/2S

where [DNA] is free DNA concentration, K_b_ is the apparent binding constant, ε_a_ corresponds to the extinction coefficient observed, and ε_f_ corresponds to the extinction coefficient of the free compound. ε_b_ is the extinction coefficient of the compound when fully bound to DNA. C_t_ is the total metal complex concentration, and S is the binding size.

### 3.4. EB Displacement Assay

The fluorescence spectrum was measured with an RF-5301 fluorescence spectrophotometer 3 mL EB-DNA solution was scanned in a colorimetric dish, and 1 mM of the complex solution was added to the solution every 3 min to measure the change of fluorescence emission peak after each addition of complex. This method measures the effect of the compound on EB fluorescence intensity of the EB-DNA system. The fluorescence spectrum of EB was measured at 520 nm excitation wavelength, and the emission range was set to 550–750 nm.

### 3.5. Molecular Docking

The molecular docking method was used to theoretically study the binding mode and binding site of aromatic Ru(II) complex in *KRAS* G-quadruplex DNA. The purpose of AutoDock 4.2 development was to solve the problem of computer-aided drug design. It is now widely used in the interaction between biological macromolecules and small-molecule ligands. The software uses Lamarck Genetic Algorithm (LGA), a semi-flexible docking method, which allows the conformation of small molecules to change, and combines free energy as the basis for evaluating the docking results. The native *KRAS* G-quadruplex sequence (5′-AGGGCGGTGTGGGAATAGGGAA-3′) within the *KRAS* proto-oncogene promoter region (PDB ID: 5I2V) and dsDNA (PDB ID: 6DY9) downloaded from the Protein Data Bank. A grid box (x = 126, y = 126, z = 126) was set to cover on *KRAS* G-quadruplex DNA, and AutoDock was used to conduct a formative search of the ligand in the box and sort the results according to the energy score. The results were analyzed by PyMol software’s. The PyMOL (1.7.4.4 Edu) Molecular Graphics System, Version 1.8 Schrödinger, LLC.

### 3.6. Cell Culture

MCF-7 human breast cancer, human triple-negative breast cancerMDA-MB-231, human esophageal cancer EC-1, and breast epithelial cells MCF-10A were purchased from American Type Culture Collection (ATCC, Manassas, VA, USA) and cultured in DMEM medium with 10% fetal bovine serum (FBS) (PAA, Pashing, Austria), penicillin (100 I.U/mL), and streptomycin (100 ng/mL) (PAA, Austria) at 37 °C in a 90% humidified incubator with 5% CO_2_.

### 3.7. MTT Assay

In order to test the in vitro anti-tumor activity of the compounds, MTT experiments were performed. Take the cells in the logarithmic growth phase and plant them in a 96-well plate at a quantity of 5 × 10^3^ cells per well, culture for 24 h, and then add gradient concentrations (0, 1.5625, 3.125, 6.25, 12.5, 25, 50, and 100 μM) of compounds. Set 3 replicate wells for each concentration sample and incubate in a CO_2_ incubator for 72 h. Add 20 μL of MTT solution to each well for 4 h, then aspirate the medium and change to 150 μL of dimethylsulfoxide. Use a microplate reader to measure the absorbance of the DMSO solution at a wavelength of 490 nm. By measuring the ability of living cells to convert MTT into blue-purple crystal formazan through succinate dehydrogenase, the number of living cells and cell viability can be judged. The data obtained was based on the average of 3 independent experiments, and the error reported was the corresponding standard deviation. The IC_50_values were calculated using commercially available software (Prism, Graphpad Software, La Jolla, CA, USA).

### 3.8. Flow Cytometric Analysis

The cells were incubated with different concentrations of **1** (0, 2.5, 5, and 10 μM) for 72 h, then trypsinized, collected the cells, and washed with PBS. In order to analyze the cell cycle arrest, the cells were fixed with 70% ethanol at 4 °C overnight. The fixed cells were washed with PBS and stained with malonimide (PI) for 5 min in the dark, and then analyzed and collected for cell cycle using Epics XL-MCL flow cytometer (Beckman Coulter, Miami, FL, USA). Data were processed using ModiFit. In order to analyze cell apoptosis, perform cell apoptosis analysis according to the protocol in the BD Pharmingen FITC Annexin-V Apoptosis Detection Kit. Add Annexin-V FITC and propidiumiodide (PI) to the 1X bingding butter cell suspension. Stain for 10 min in the dark. Then, the Epics XL-MCL flow cytometer (Beckman Coulter, Miami, FL, USA) was used to collect data and perform apoptosis analysis.

### 3.9. Comet Assay

MCF-7 cells (5 × 10^4^ cell/well) were seeded in 6-well plates containing 0, 2.5, 5, and 10 μM compound **1**, respectively, and cultured for 72 h, and cells of each treatment group were collected. PBS and 1% low melting point agar gel were mixed with the cells at a ratio of 1:8; drop the cell suspension on the comet glass slide and cover it with a cover glass. After solidification, remove the cover glass for lysis for 1 h above at 4 °C. After washing with PBS, place the slides in the electrophoresis solution for unspinning, cover with a cover glass 20 min later, and run electrophoresis at 25 V, 250 mA for 30 min. After the electrophoresis was completed, the cover glass was removed and stained in a 1 mg/mL EB solution at 4 °C for 20 min in the dark. After washing with PBS, the cover glass was covered and photographed with a fluorescence microscope.

### 3.10. Immunofluorescence

For immunofluorescent staining, cells were fixed in 4% paraformaldehyde for 20 min, permeabilized with 0.5% Triton and blocked with 1% BSA for 60 min at 37 °C. The cells were incubated with primary antibodies overnight at 4 °C, washed 3 times in PBST, and then incubated with Alexa Fluor 488-conjugated secondary antibodies for 60 min. DNA was counterstained with 1 g/mL DAPI for 15 min at 37 °C. Cells mounted on coverslips were observed with a ZEISS LSM 800 confocal laser scanning microscope.

## 4. Conclusions

In the present study, three arene Ru(II) complexes coordinated with different substituent phenanthroimidazole derivatives were synthesized under microwave irradiation and studied for their ability to stabilize *KRAS* G-quadruplex DNA and inhibit the proliferation of breast cancer cells. The chlorine substitution site on the ligand plays an important role in improving the DNA interaction of this class of Ru(II) metal complexes. Spectroscopy and molecular docking has shown that arene Ru(II) complexes, particularly the ortho-para substitution ones **1** and **3**, were effective and selective stabilizers of G4 motifs present in the *KRAS* promoter. Cell activity assay confirms that the derivatives **1** will significantly reduce the proliferation of MCF-7 in the micromolar concentration range. However, the cytotoxicity of this class of compounds in 3D cell models is still unclear. This will be carried out in the next study. Further studies reveal that **1** can induce G0/G1 phase arrest and apoptosis through DNA damage mediated pathway. Such studies will be useful to identify and develop suitable lead derivatives which have specific interaction with *KRAS* G-quadruplex DNA and exhibit potential anti-cancer activity.

## Figures and Tables

**Table 1:** Inhibitory activity (IC_50_/μM) of the Ru(II) complexes against selected cell lines.

Comp.	Inhibitory Activity (μM)			
	MDA-MB-231	MCF-7	EC-1	MCF-10A
**1**	>100	3.7 ± 0.2	70.9 ± 5.0	>100
**2**	92.8 ± 3.8	>100	75.2 ± 6.3	>100
**3**	43.6 ± 1.3	>100	>100	>100
Cis-platin	58.4 ± 1.0	7.9 ± 0.2	-	33.2 ± 0.9

**Table 2:** Comparison of intracellular accumulation of ruthenium after treatment with complexes **1**, **2** and **3**.

Comp.	Ruthenium Uptake (μg L^−1^, 4 h)		
	MDA-MB-231	MCF-7	MCF-10A
**1**	0.645	3.438	3.674
**2**	5.268	5.531	5.730
**3**	3.674	1.018	1.995

Note: Cells treated with 20 μM concentrations of each complex.

## Data Availability

Dates of the compounds are available from the authors.

## Supplementary Materials

The following supporting information can be downloaded at: https://www.mdpi.com/article/10.3390/molecules27103046/s1, Figure S1: ESI-MS of Ru(II) complexes **1**; Figure S2: 1H-NMR of Ru(II) complexes **1**; Figure S3: ^13^C-NMR of Ru(II)complexes **1**; Figure S4: ESI-MS of Ru(II) complexes **2**; Figure S5: ^1^H-NMR of Ru(II) complexes **2**; Figure S6: ^13^C-NMR of Ru(II) complexes **2**, Figure S7: ESI-MS of Ru(II) complexes **3**; Figure S8: ^1^H-NMR of Ru(II) complexes **3**; Figure S9: ^13^C-NMR of Ru(II) complexes **3**; Figure S10: The HPCL results of complex **1**, the mobile phase is CH_3_OH:CH_3_CN=8:2; Figure S11: The HPCL results of complex **2**, the mobile phase is CH_3_OH:CH_3_CN=8:2; Figure S12: The HPCL results of complex **3**, the mobile phase is CH_3_OH:CH_3_CN=8:2; Figure S13: Binding site and mode of **1** (cyan), **2** (green), **3** (yellow) with c-myc (A), Bcl-2 (B) and VEGF (C) G-quadruplex DNA calculated by molecular docking; Figure S14: CD titration spectra of *KRAS* G-quadruplex DNA (2 μM) at different concentrations of **1** (A), **2** (B) and **3** (C) ([Ru] = 0, 8, 16, 24 and 32 μM) in the incubation buffer; Figure S15: (A) The replication blocking of PCR products through stabilizing *KRAS* G-quadruplex DNA treated with arene Ru(II) complexes. (B) Effect of complexes **1**, **2**, and **3** on the PCR-stop assay with *KRAS* G-quadruplex DNA. [Ru] = 0, 0.625,1.25, 2.5, 5, 10 and 20 μM, [*KRAS*] = 10 pM. Figure S16: The stability of *KRAS* G-quadruplex DNA induced by arene ruthenium(II) complexes.

## Sample Availability

Samples of the compounds are not available from the authors.
