# Supplementary material for: Arene Ru(II) Complexes Acted as Potential KRAS G-Quadruplex DNA Stabilizer Induced DNA Damage Mediated Apoptosis to Inhibit Breast Cancer Progress
Source: Molecules. 2022 May 10;27(10):3046. doi: 10.3390/molecules27103046 (PMC9146995; doi:10.3390/molecules27103046)
Supplement: Supplementary file 1 [file molecules-27-03046-s001.zip › molecules-1608425-supplementary.pdf]

Supporting Information

Arene Ru(II) complexes acted as potential *KRAS* G-quadruplex DNA stabilizer induced DNA damage mediated apoptosis to inhibit breast cancer progress

Jiayi Qian[1], Ruotong Liu[1], Ningzhi Liu[1], Chanling Yuan[1], Qiong Wu[2], Yanhua Chen[1], Weijun Tan[2,3,*], and Wenjie Mei[1,2,*]

1. School of Pharmacy,Guangdong Pharmaceutical University, Guangzhou 510006, China; qianjiayi0101@163.com (J.Q.); ruotongliu123@126.com (R.L.); ganoderma0@126.com (N.L.); ycc2552@163.com (C.Y.); amandachen423@163.com (Y.C.)

2. Guangdong Province Engineering Technology Centre for Molecular Probes and Biomedicine Imaging, Guangzhou, 510006, China; wuqiongniu.1113@163.com

3. School of Food Science, Guangdong Pharmaceutical University, Guangzhou, 51006, China

Correspondence: tanwj@gdpu.edu.cn (W.T.); wenjiemei@gdpu.edu.cn (W.M.)

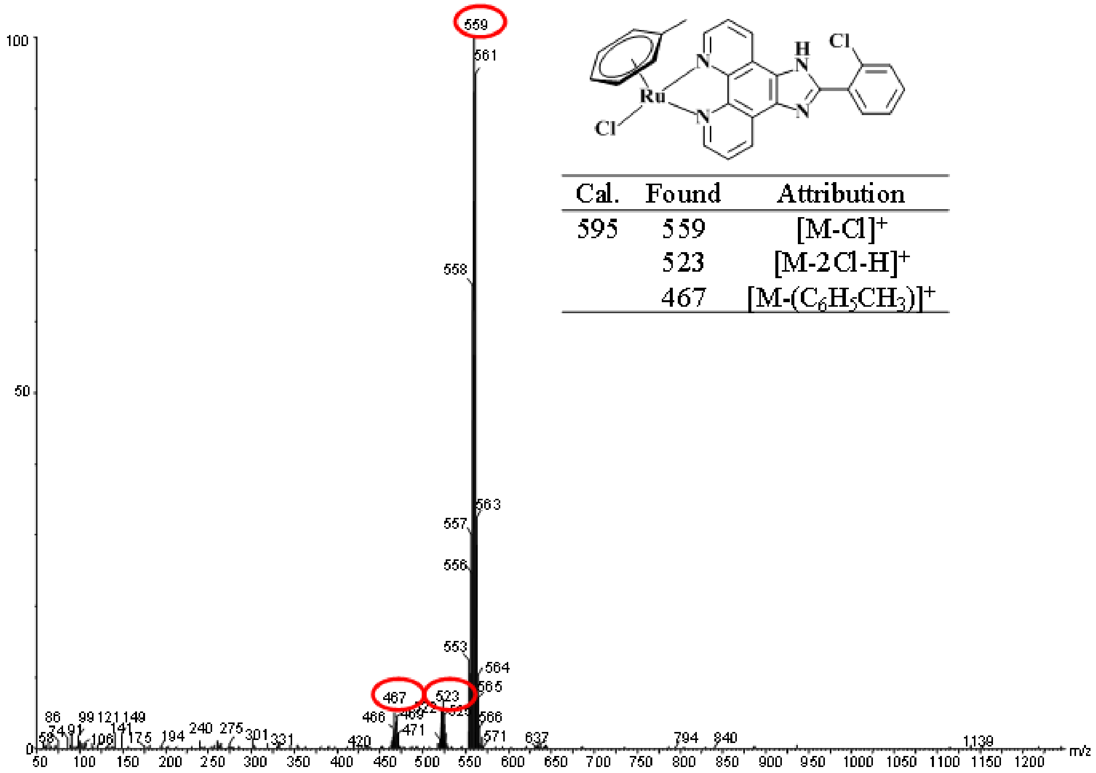

Cal.	Found	Attribution
595	559	[M-Cl]$^+$
	523	[M-2Cl-H]$^+$
	467	[M-($C_6H_5CH_3$)]$^+$

Figure S1. The ESI-MS spectra of complex **1.**

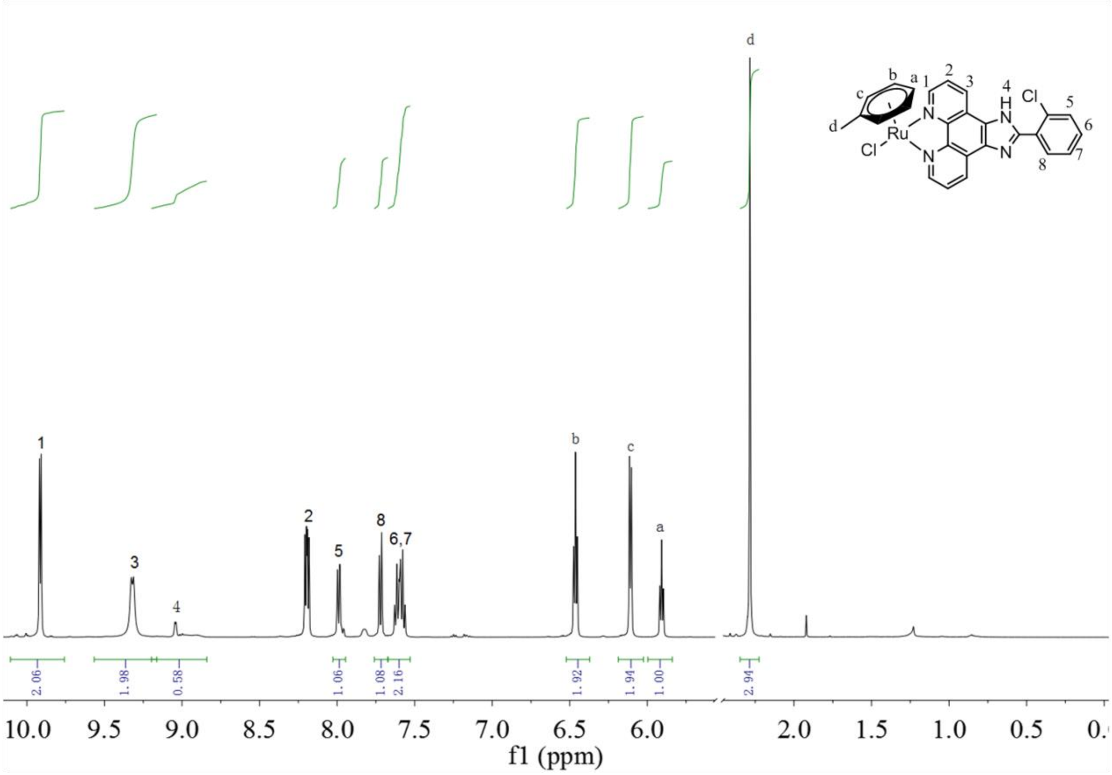

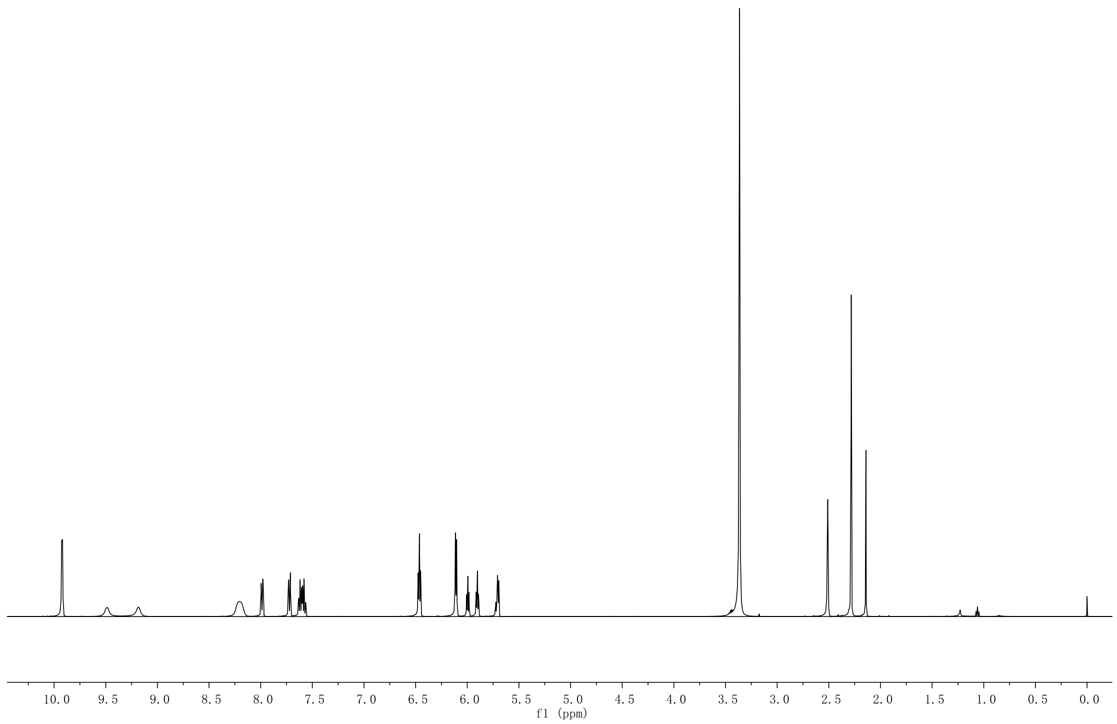

Figure S2. The partial enlarged view (A) and the whole view (B) of ¹H NMR spectra of complex **1.**

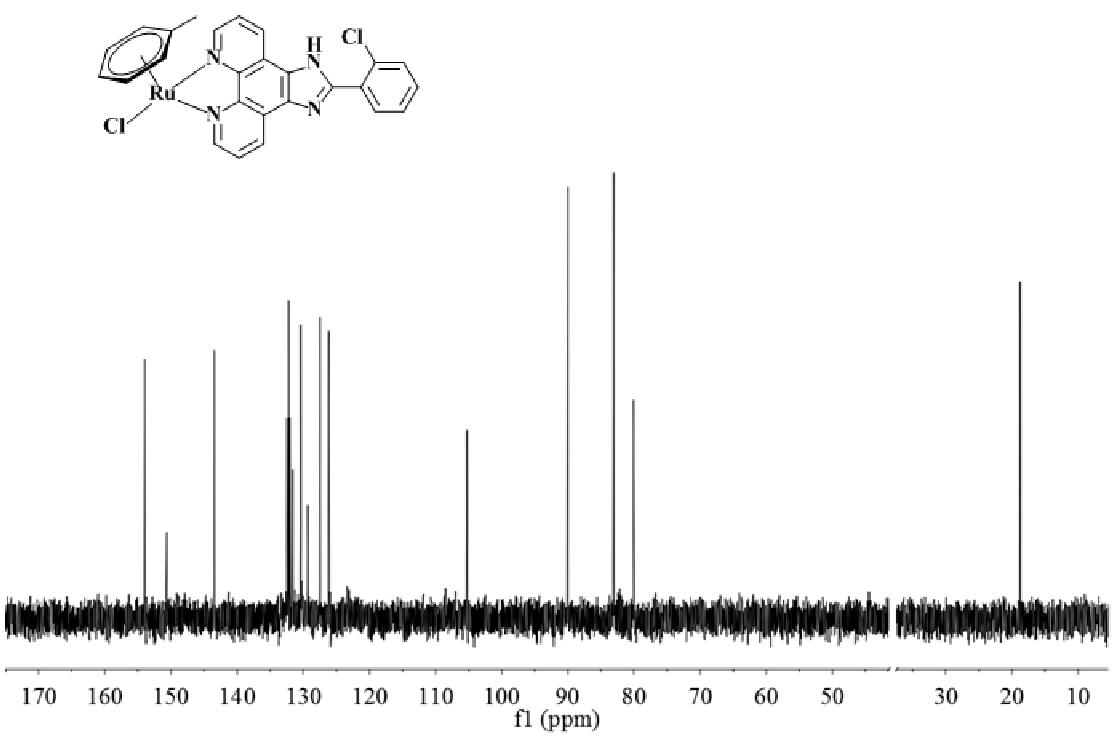

Figure S3. The ^{13}C NMR spectra of complex **1.**

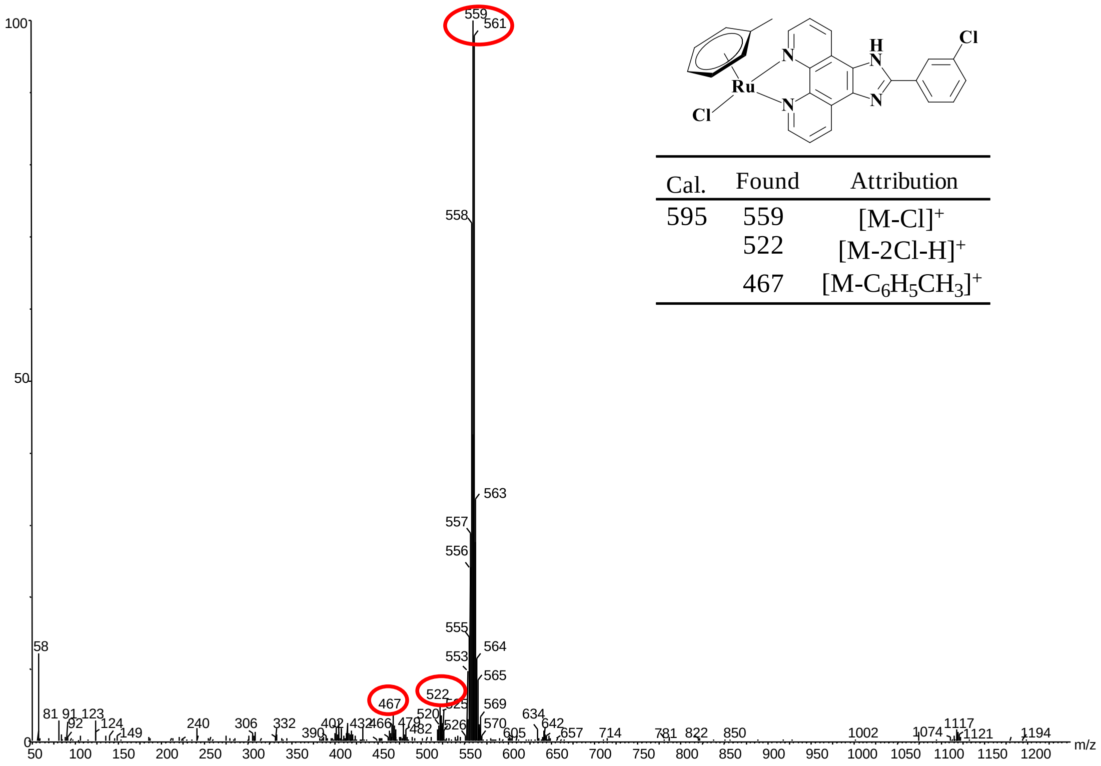

Cal.	Found	Attribution
595	559	$[M-Cl]^+$
	522	$[M-2Cl-H]^+$
	467	$[M-C_6H_5CH_3]^+$

Figure S4. The ESI-MS spectra of complex **2.**

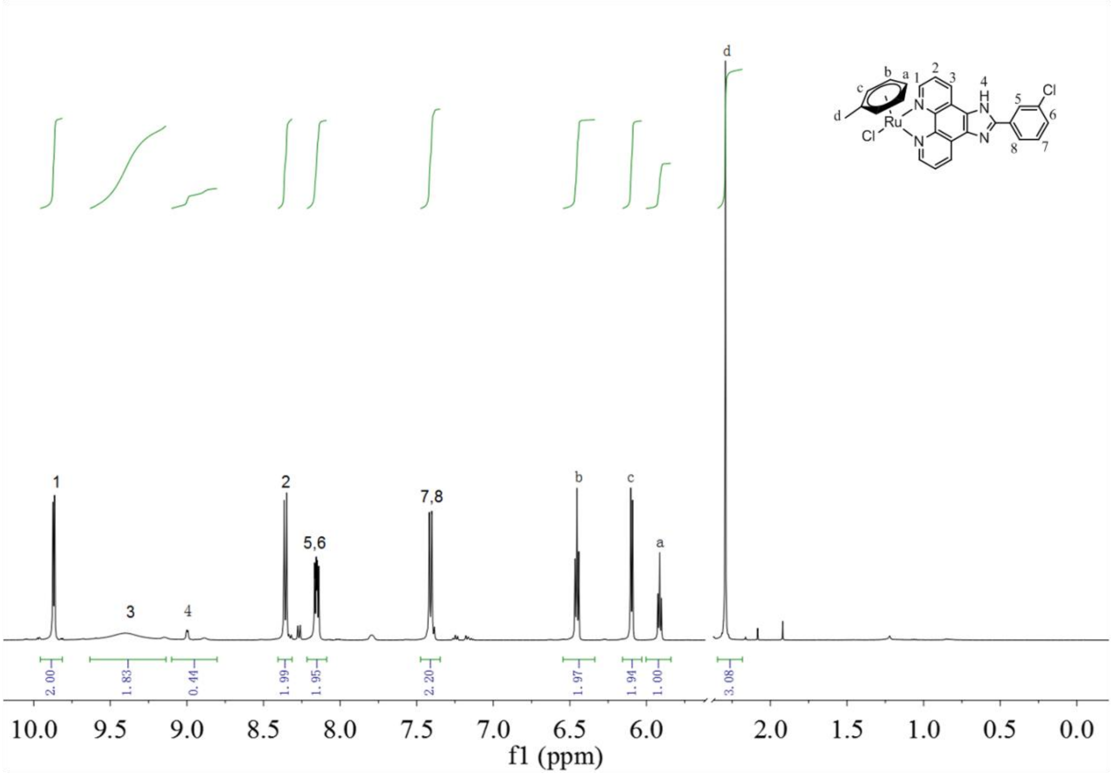

A

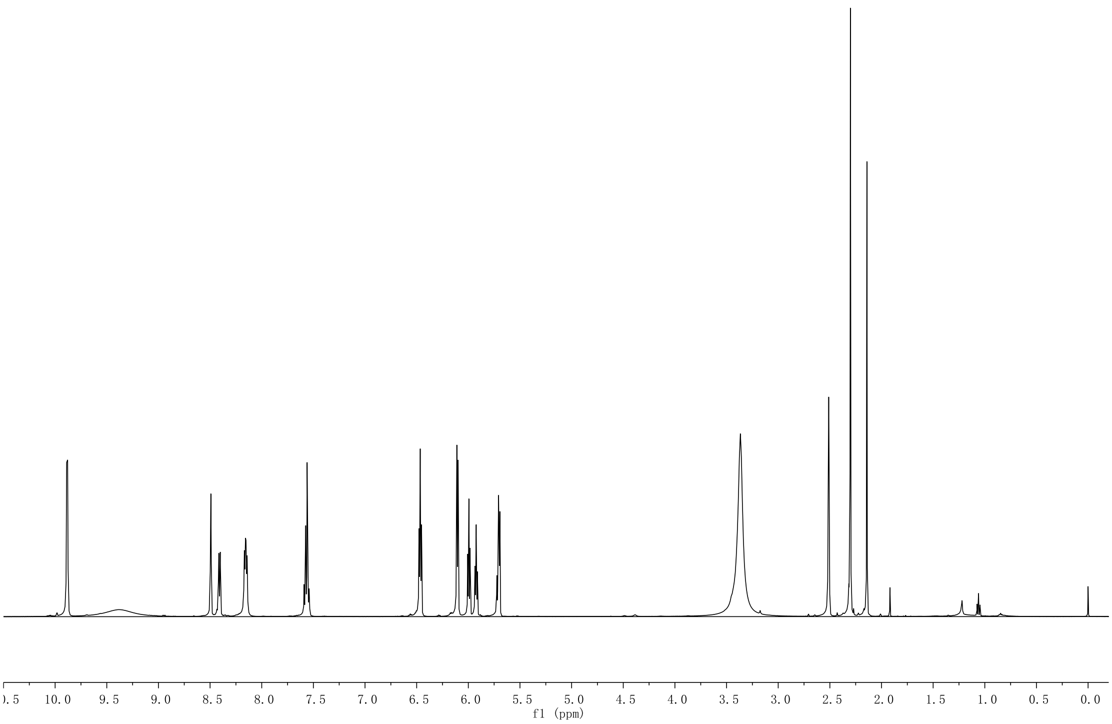

B

Figure S5. The partial enlarged view (A) and the whole view (B) of ^{1}H NMR spectra of complex **2.**

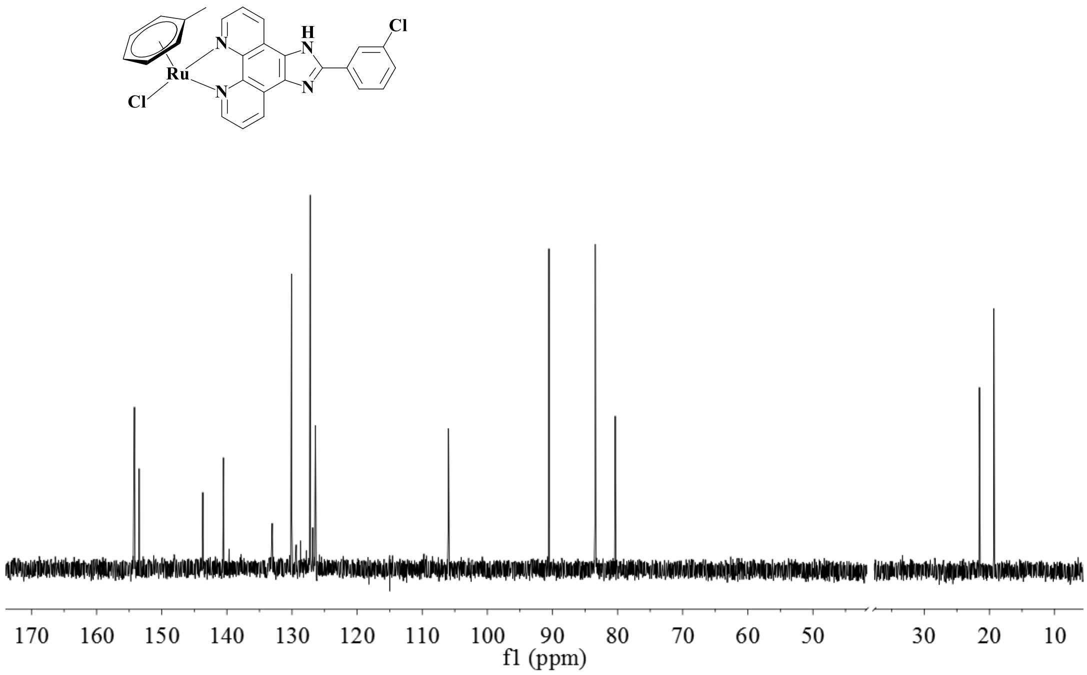

Figure S6. The ^{13}C NMR spectra of complex **2.**

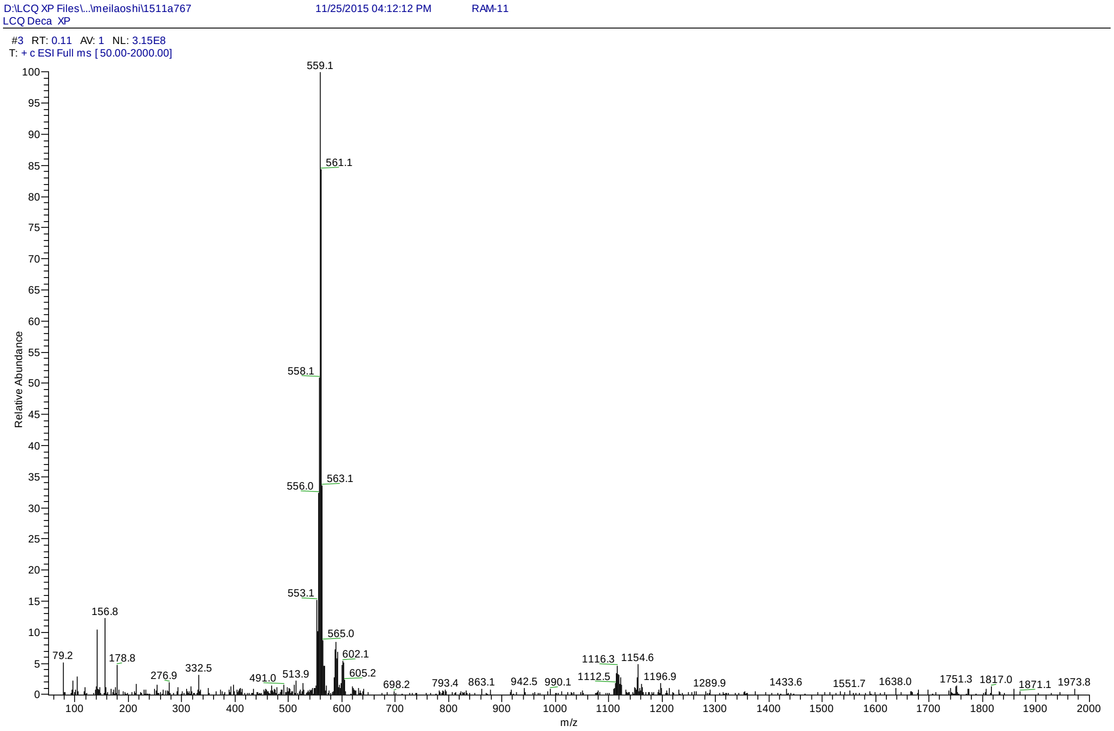

Figure S7. The ESI-MS spectra of complex **3.**

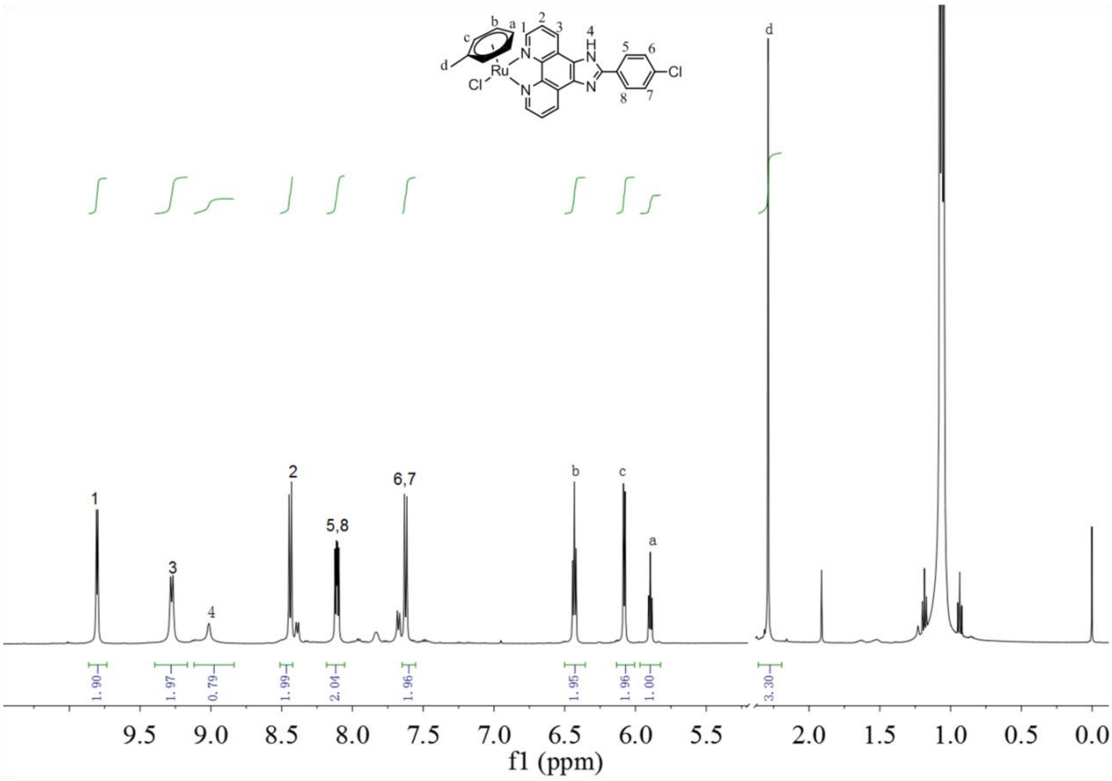

A

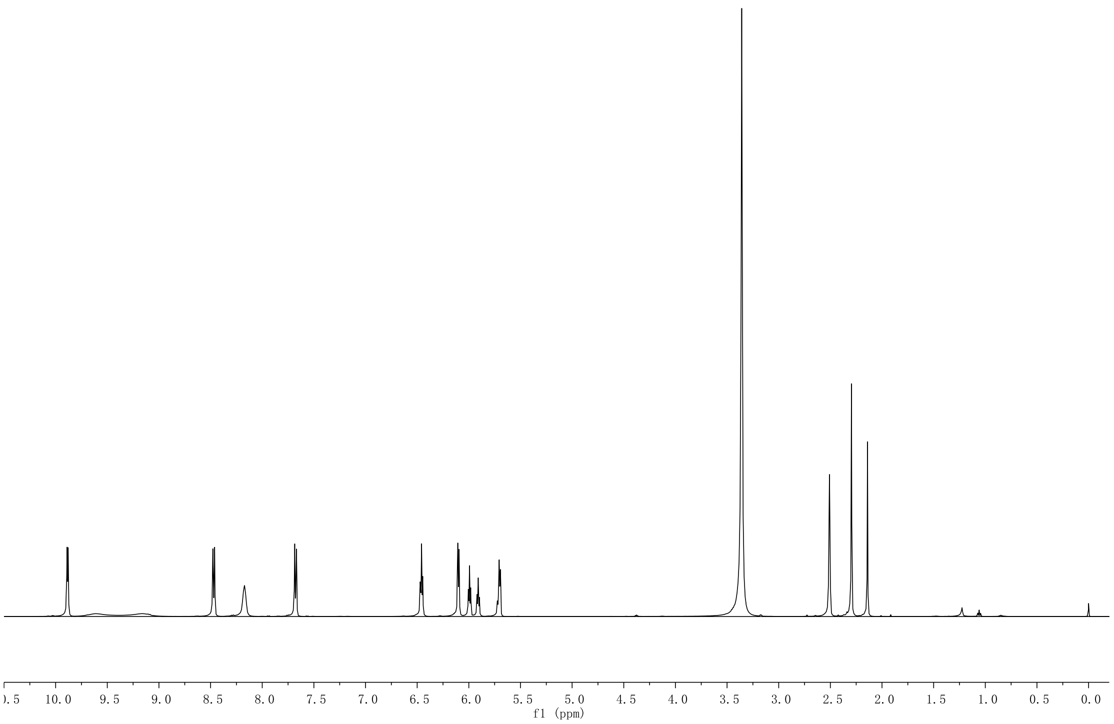

B

Figure S8. The partial enlarged view (A) and the whole view (B) of ^{1}H NMR spectra of complex **3.**

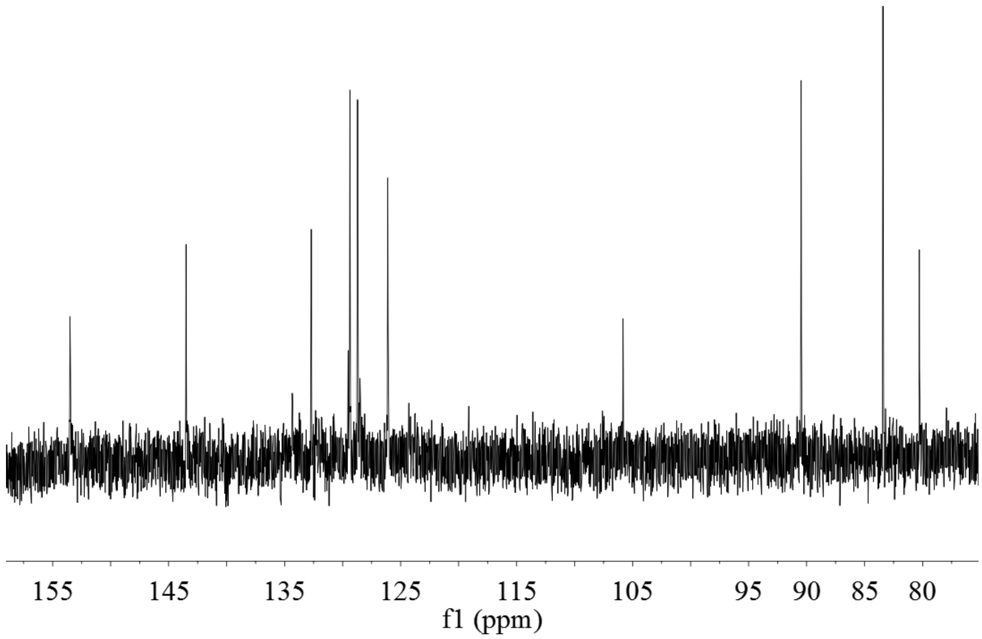

Figure S9. The ^{13}C NMR spectra of complex **3.**

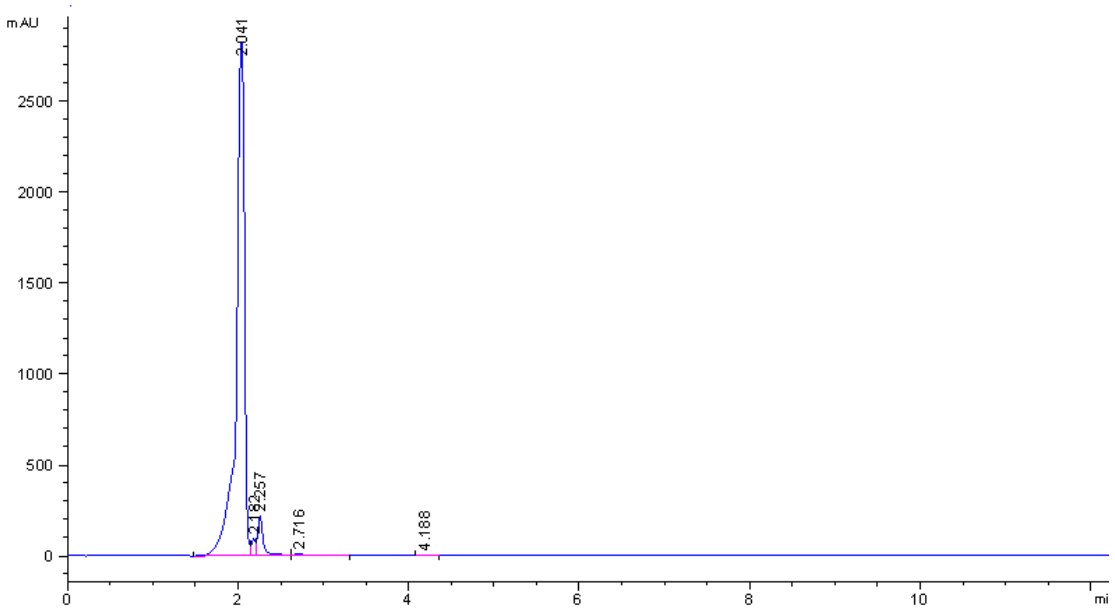

Figure S10. The HPCL results of complex **1**, the mobile phase is CH_3OH:CH_3CN=8:2.

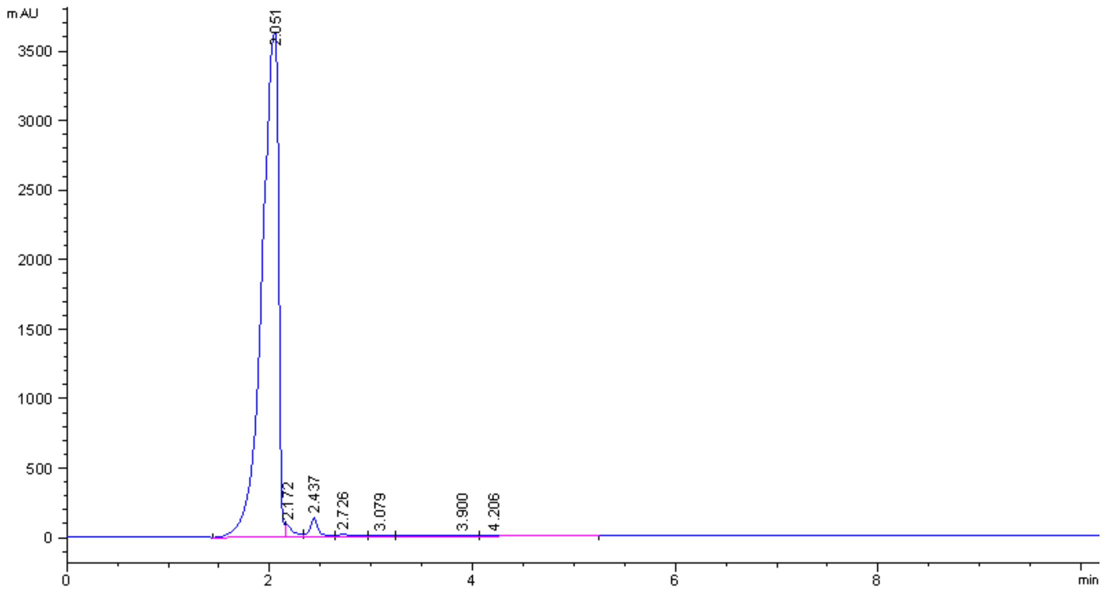

Figure S11. The HPCL results of complex **2**, the mobile phase is CH_3OH:CH_3CN=8:2.

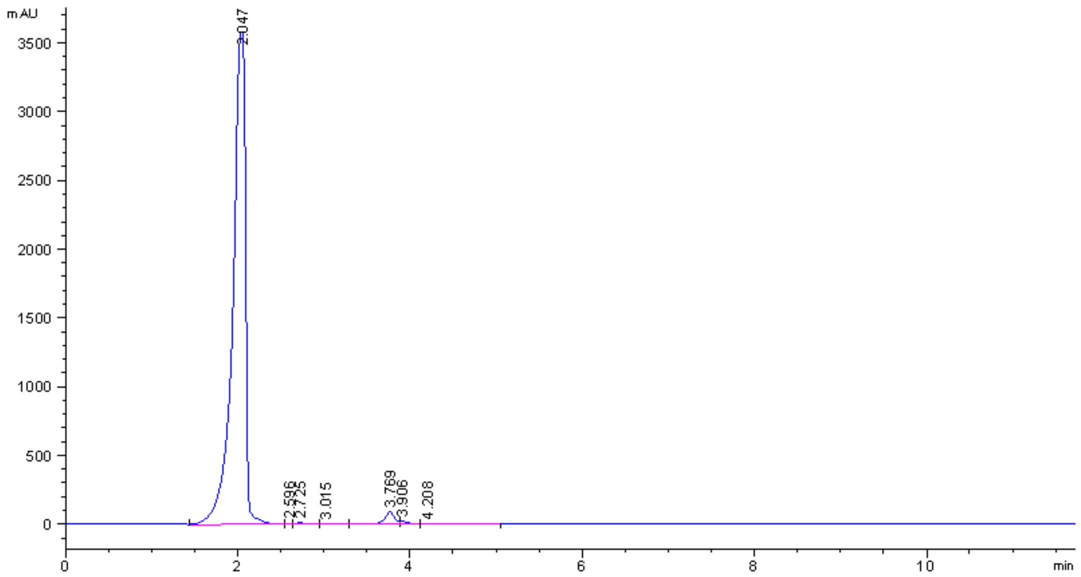

Figure S12. The HPCL results of complex **3**, the mobile phase is CH_3OH:CH_3CN=8:2.

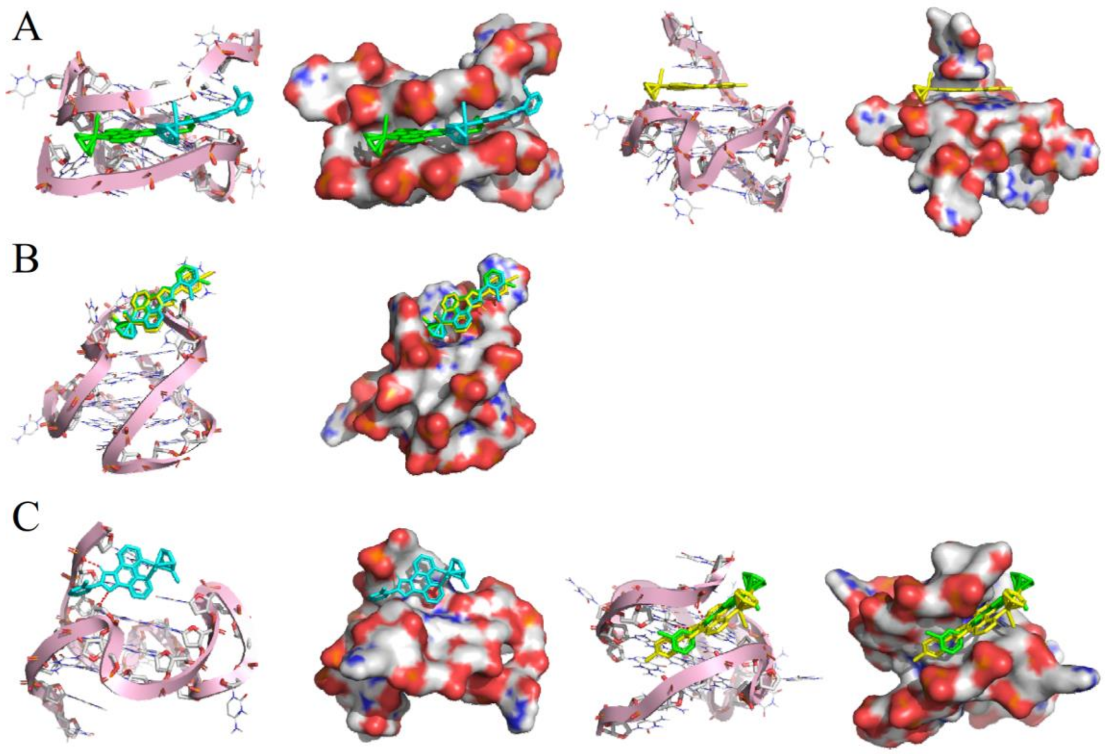

Figure S13. Binding site and mode of **1**(cyan), **2**(green), **3**(yellow) with c-myc (A), Bcl-2 (B) and VEGF (C) G-quadruplex DNA calculated by molecular docking.

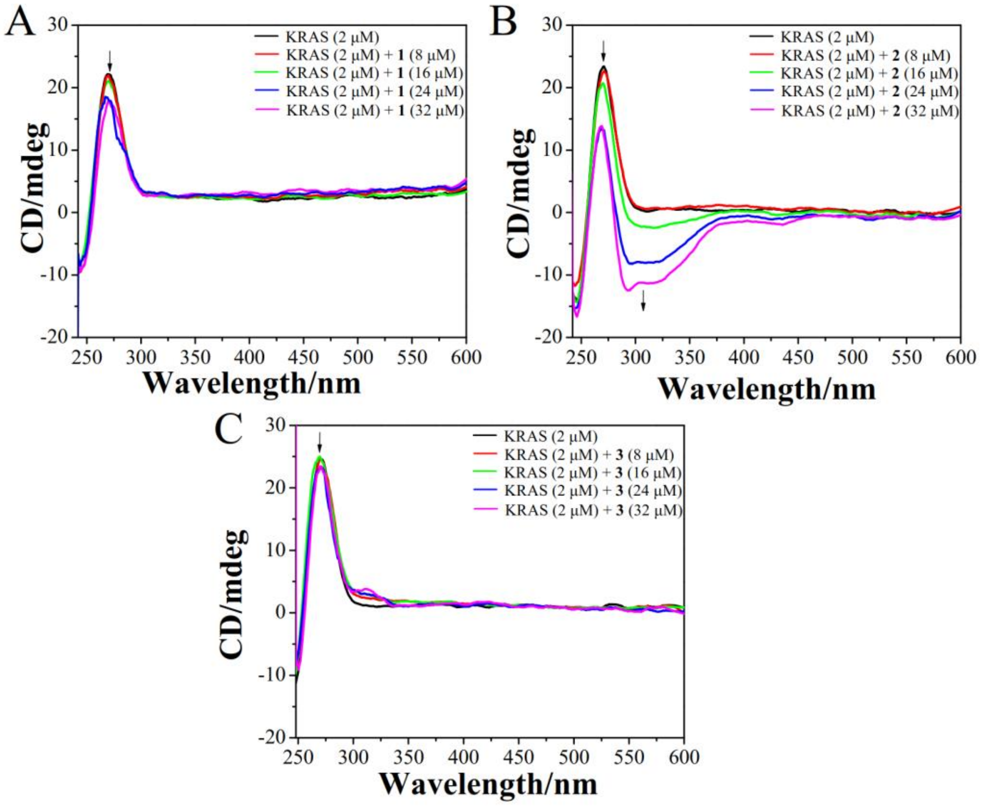

Figure S14. CD titration spectra of KRAS G-quadruplex DNA (2 μM) at different concentrations of **1** (A), **2** (B) and **3** (C) ([Ru] = 0, 8, 16, 24 and 32 μM) in the incubation buffer.

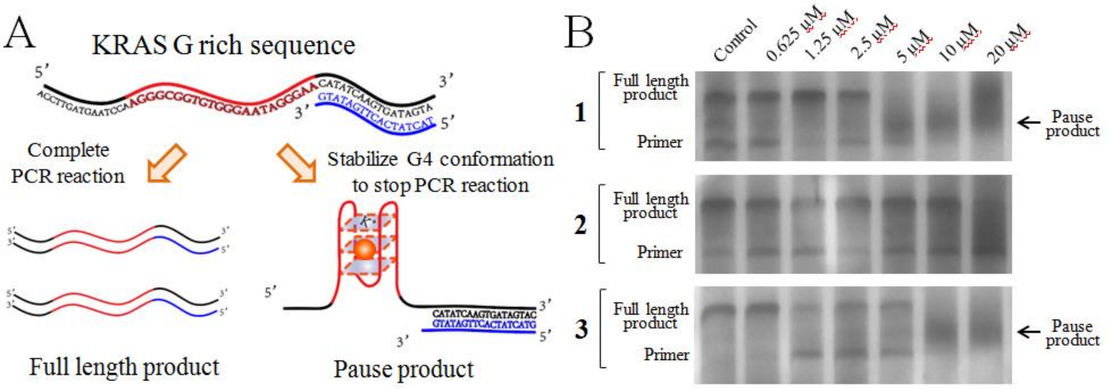

Figure S15. (A) The replication blocking of PCR products through stabilizing KRAS G-quadruplex DNA treated with arene Ru(II) complexes. (B) Effect of complexes **1**, **2** and **3** on the PCR-stop assay with KRAS G-quadruplex DNA.

[Ru] = 0, 0.625,1.25, 2.5, 5, 10 and 20 μM, [KRAS] = 10 pM.

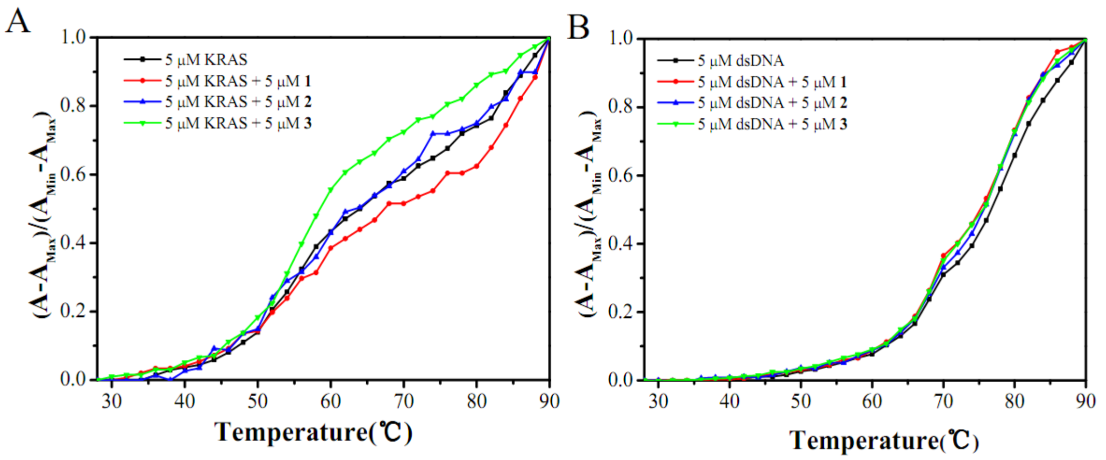

Figure S16. The stability of KRAS G-quadruplex DNA induced by arene ruthenium(II) complexes. (A) UV melting curves obtained with KRAS G-quadruplex DNA (5 μM) free (■) upon addition of **1** (◆), **2** (▲) and **3** (▼). (B) UV melting curves obtained with dsDNA (5 μM) free (■) upon addition of **1** (◆), **2** (▲) and **3** (▼). [Ru]= 5 μM.
